# Rate of Retained Surgical Foreign Bodies in Texas Medicare Beneficiaries: Post-Pandemic Analysis

**DOI:** 10.7759/cureus.82314

**Published:** 2025-04-15

**Authors:** Daphne E Sanchez, Jay C Wang, Gisela M Ortega, Rebecca L Sanchez

**Affiliations:** 1 Department of Applied Biomedical Sciences, University of the Incarnate Word School of Osteopathic Medicine, San Antonio, USA

**Keywords:** covid-19, foreign object, sars-cov-2, surgical bodies, texas

## Abstract

Objective: This study aimed to identify the difference between the rates of retained surgical foreign bodies (RSBs) in Texas Medicare beneficiaries before and after the COVID-19 pandemic, 2017-2019 and 2020-2022, respectively, by county and by the Human Health Service Commission (HHSC) region.

Background: Retained surgical foreign bodies (RSBs) are items left in patients’ bodies after surgical interventions (e.g., sponges, surgical instruments, etc.). Studies have shown an association between an increased risk of RSBs and unexpected intraoperative events, procedure duration, incorrect surgical counts, and variations in personnel on surgical teams. However, the existing literature has not focused on the impact of SARS-CoV-2 or the COVID-19 pandemic on RSB rates.

Methods: Data from the Centers for Medicare and Medicaid Services Datasets from 2020-2023 were used, along with Texas Hospital Data from the Texas Department of State and Health Services, to categorize the mean rates of RSBs before and after the COVID-19 pandemic by Texas HHSC regions and counties.

Results: No significant differences were found between the pandemic-era and HHSC regions. However, the differences between Texas counties before and after the COVID-19 pandemic were statistically significant for both Colorado and Victoria. All other Texas counties showed no significant changes before and after the COVID-19 pandemic.

Conclusion: Given the importance of reducing RSBs, follow-up studies that review specific surgical policies before and after the COVID-19 pandemic should be conducted.

## Introduction

Retained surgical foreign bodies (RSBs) are items left in the bodies of patients after surgical interventions (e.g., sponges and surgical instruments). In the United States (U.S.), more than 28 million surgical procedures are performed per year, with an estimated 1,500 cases of RSBs [1]. RSBs may lead to additional hospital-acquired conditions that range from systemic symptoms, such as fever and fatigue, to potentially life-threatening complications, such as abscesses and obstructive processes, throughout a variety of organ systems [1]. Studies have found that unexpected intraoperative events, procedure duration, incorrect surgical counts, and variations in personnel in surgical teams increase the risk of RSBs [2]. 

During the COVID-19 pandemic, U.S. healthcare systems were severely overwhelmed, leading to massive disruptions in usual care delivery patterns, including understaffing and shortages in personal protective equipment and other medical supplies [3,4]. These concerns led the American College of Surgeons and other surgical specialty societies to recommend minimizing elective surgical procedures and triaging emergent cases [4]. Concurrently, the Centers for Medicare & Medicaid Services and the U.S. Surgeon General released similar statements on postponing nonessential operations [4]. 

After the COVID-19 pandemic, significant strain was placed on healthcare teams, where providers faced burnout and others left the profession, including surgical trainees [5]. The decline of healthcare teams, operational strains, and patient “spillover” of non-COVID-19 patients led to a backlog of surgeries and disruption of overall care [3,6]. These disruptions were particularly evident in healthcare settings that serve vulnerable populations, such as communities of color and people insured by Medicare [3]. 

RSBs demonstrate a failure on the part of the surgical team as a whole to guarantee patient safety despite the many measures that exist in the operating room to prevent them [2]. Significant increases in hospital RSB rates may highlight the potential structural failures of a facility to adapt to internal or external stressors [2,7,8]. Decreased rates, on the other hand, may demonstrate successful organizational adjustments that allow for performance variability within rapidly changing conditions [7]. This study aimed to identify any differences in mean rates of RSBs in Texas Medicare beneficiaries before and after the COVID-19 pandemic in 2017-2019 and 2020-2022 by county and by the Human Health Service Commission (HHSC) region, respectively. Identifying any difference, including both increases and decreases, in the mean rates for RSBs during this timeframe may help to understand how patient safety during surgery is impacted during times of crisis (e.g., pandemics) and guide further research on how to prevent related sentinel events.

## Materials and methods

Publicly accessible data from 2020 to 2023 were used from the Deficit Reduction Action (DRA) Hospital-Acquired Conditions (HAC) Measures from the Centers for Medicare and Medicare Services and the Texas Hospital Data from the Texas Department of State and Health Services [9,10]. The DRA-HAC dataset reported mean observed rates per 1,000 discharges within 18 to 24 months of the reporting periods and reported factors such as foreign objects retained after surgery, blood incompatibility, air embolism, falls, and trauma [9]. The Texas Hospital Dataset reported factors such as facility and bed counts, charity care, outpatient and emergency department visits, admissions, inpatient days, length of stay, and surgical operations [10]. Data were filtered to include only “Foreign Object Retained After Surgery” from the DRA-HAC to determine RSB rates. Hospital location and provider reporting rates were associated with listed Medicare and Medicare Services Provider IDs. Data points were then divided according to their associated Texas HHSC region via provider identification numbers and their respective county. To establish chronologicity with the data, reports with an end quarter before 2020 were categorized as “pre-COVID,” while reports with a start quarter of 2020 or after were reported as “post-COVID.” Data excluded from the finalized dataset included provider IDs outside of Texas and any data point containing “Footnote 23," which indicated provider-reported discrepancies. 

Statistical analysis 

All data were analyzed using R, an open-source programming software used for statistical analysis, version 4.3.2. Welch’s two-sided t-test was conducted to determine the difference in means of RSB rates between the pre-COVID and post-COVID groups. Two-way analysis of variance (ANOVA) interaction tests were performed to determine the difference in means of RSB rates between all HHSC regions from 2020 to 2023 and the difference in means of RSB rates between pre-COVID and post-COVID periods in HHSC regions. Two-way ANOVA interaction tests were also used to determine the difference in means of RSB rates among all Texas counties and the difference in means of RSB rates between pre-COVID and post-COVID periods of Texas counties. Following any ANOVA analysis demonstrating a significant difference (p-value < 0.05), Tukey post hoc analyses were performed to identify variables with significantly different mean RSB rates.

## Results

The mean RSB rate in Texas during the pre-COVID years (M = 0.0165) was greater than the mean RSB during the post-COVID years (M = 0.0090). However, this difference was not statistically significant (t = -0.94216, df = 404.04, p = 0.3467).

**Figure 1:**
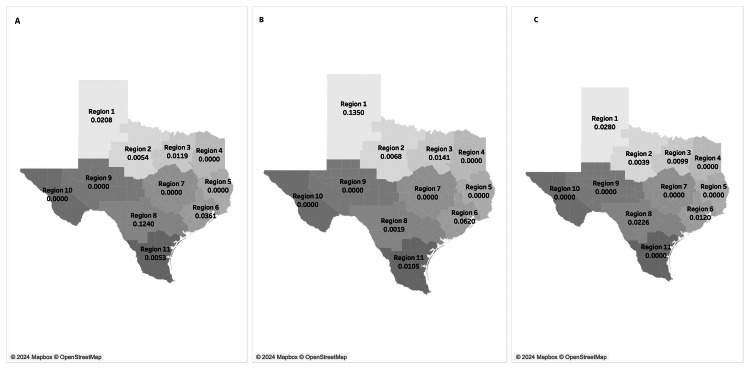
Average rates of RSB by Texas HHSC region overall, pre-COVID, and post-COVID Visual representation of Texas HHS Regions and each region’s mean RSB rates for (A) the 2017 to 2022 study period overall, (B) pre-COVID years, and (C) post-COVID years.  No significant difference was determined by HHS region for overall, pre-COVID, and post-COVID eras. The map of Texas HHS regions were digitally created by Tableau using an open-source map of Texas (i.e., open maps) with regional coordination of Texas counties from Texas Health and Human Services [10]. Average rates were calculated by the authors and superimposed on the digital image. HHS: Health and Human Services, RSB: Retained Surgical Foreign Bodies.

The HHSC region 6 had the highest mean RSB rate (M = 0.0361). HHSC regions 1, 8, 3, 2, and 11 followed in descending order, as shown in Figure 1A. However, the difference in the mean rates of RSB by HHSC region was not statistically significant (F-value = 0.202, p = 0.653). HHSC regions 4, 5, 7, 9, and 10 showed no overall observed rates (M = 0.0000). The mean RSB rates per HHSC region during pre-years and post-COVID years were also analyzed. HHSC region 6 had the greatest average difference in RSB pre-COVID (M = 0.0620) compared with post-COVID (M = 0.0120). Changes in mean RSB rates from pre-COVID to post-COVID years were observed in HHSC regions 1, 2, 3, 8, and 11 (Figures 1B, 1C). There was no significant difference between HHSC regions and the COVID era (pre-/post-COVID) (F-value = 0.117, p = 0.732).

The mean RSB rates by county were compiled (Table 1) and demonstrated that Colorado, Potter, and Victoria Counties had the greatest average rates for all years (M = 0.8678, 0.1503, and 0.1473, respectively). An ANOVA analysis of mean RSB rates for all years determined significant variation among Texas counties (F-value = 16.524, p-value < 0.05). When compared to other Texas counties, the post hoc Tukey test showed that Colorado County was significantly different (p < 0.05). The mean RSB rates in Colorado County were also statistically significant when compared to every other county individually (p < 0.05). An ANOVA interaction regarding the counties during the pre- and post-COVID eras determined significant variation (F-value = 16.360, p-value < 0.05). A post-hoc Tukey test showed that Colorado pre-COVID rates and Victoria post-COVID rates differed significantly when individually compared to every other county’s pre-COVID and post-COVID rates at p < 0.05. Notably, Colorado rates significantly decreased between pre- and post-COVID years, while Victoria rates significantly increased (p < 0.05).

**Table 1:** Average rates of foreign objects retained after surgery by Texas counties and COVID era (ANOVA) Mean and standard deviation RSB rates of each county were determined for (1) the 2017 to 2022 study period overall, (2) pre-COVID years, and (3) post-COVID years. A two-way ANOVA interaction test was performed to determine the difference in RSB rate means between counties and pre-COVID and post-COVID years. Following any ANOVA analysis that demonstrated a significant difference, a Tukey post-hoc analysis was performed to identify which means carried a statistical significance of a p-value of < 0.05. Texas counties with a value of 0.000 for all three categories were excluded from the table.

County	Overall Mean	Overall Std. Dev.	Mean Pre-COVID	Std. Dev. Pre-COVID	Mean Post-COVID	Std. Dev. Post-COVID
Bexar	0.005	0.0136	0.004	0.0103	0.006	0.0165
Cameron	0.0369	0.104	0.0738	0.148	0.000	0.000
Collin	0.0534	0.0899	0.0403	0.0810	0.0665	0.0998
Colorado	0.868*	1.0508	1.736*	0.548	0.000	0.000
Dallas	0.005	0.0188	0.003	0.0149	0.0068	0.0223
Denton	0.0275	0.0987	0.0595	0.142	0.000	0.000
Harris	0.0100	0.0562	0.000	0.000	0.0189	0.0768
Lubbock	0.0398	0.0687	0.0255	0.0402	0.0542	0.0910
Potter	0.150	0.101	0.099	0.140	0.202	0.0205
Victoria	0.147	0.171	0.000	0.000	0.295*	0.0276
Wichita	0.0349	0.0488	0.0443	0.0523	0.0255	0.051

## Discussion

Due to the increased risk of abscesses and obstructive processes throughout various organ systems from RSBs, patients should immediately contact their surgeons should complications occur [1]. However, for Medicare beneficiaries, a particularly vulnerable population with healthcare barriers (e.g., transportation, finances, technology), appropriate follow-up with their providers may not be typically seen [11,12]. This is concerning, as postoperative complications most commonly occur after hospital discharge, taking weeks or months to manifest adverse reactions and, less commonly, years [2]. Combining these health disparities with emergent situations, such as the COVID-19 pandemic, further increases the risk of surgical complications like RSBs, and as such, prevention is key. 

As RSBs are considered completely preventable events, their occurrence signals a breakdown in surgical safety protocols and reflects systemic vulnerabilities within the operating room team and hospital infrastructure [1,2]. Significant fluctuations in RSB rates can serve as indicators of a hospital’s ability, or inability, to adapt and offer insight into the performance variability of a hospital during times of crisis. Performance variability is the ability of healthcare systems and providers to adjust their policies and daily tasks to maximize productivity and the safety of patients [7]. When existing systems are pushed to their limits or presented with novel scenarios, they can either adjust accordingly to expand their previously existing capacities or fail in their adaptability, resulting in productivity and system failure [8]. Another way to consider this is in terms of long-term, sustainable adjustments versus short-term, reactionary system adaptations. For COVID-19, examples of long-term adjustments included expansions in the scope of telehealth and altered procedures (e.g., use and conversion of available space, updated personal protective equipment, hiring more personnel, and alternative triage and patient assessment procedures) [7]. Short-term, reactionary adaptations are more centered on individual behaviors rather than systemic changes, and thus, they conceal structural and operational problems instead of solving them. These reactionary solutions by individuals include actions like employees working more hours, increasing workload, and training for and taking on additional responsibilities [13]. In a surgical context, these short-term adaptations can include extending operating room hours or scheduling weekend cases [14]. While necessary at the moment, short-term adaptations are not sustainable and, without organizational change, risk an unexpected decline in performance, which increased RSB rates may illustrate [8].

Physician burnout was another concern resulting from the failure of performance variability, such as high workloads, persistent understaffing, subsequent high levels of workforce turnover, and shifting expectations and scope of provider roles (e.g., increased patient education and social and emotional support for end-of-life patients) [7,15,16]. This additional prevalence of burnout for physicians was concerning, particularly for surgeons, who have been found to have an increased rate of burnout compared to other medical specialists [16-18]. Studies have found that physician burnout creates additional mental and emotional strain on providers, causing decreased performance, poor communication, and deterioration of teamwork, which can subsequently lead to poor patient safety outcomes [18].

The absence of statistically significant changes in RSB rates across most Texas counties, apart from Colorado and Victoria counties, suggests that the majority of Texas hospitals successfully adapted to the operational and clinical challenges introduced by the COVID-19 pandemic. Despite the well-documented strains on healthcare systems, most institutions maintained stable RSB rates, indicating effective procedural safeguards and system resilience. Additionally, all but the eleven Texas counties listed in Table 1 (Bexar, Cameron, Collin, Colorado, Dallas, Denton, Harris, Lubbock, Potter, Victoria, and Wichita) had a rate of 0.000 RSBs for the overall study period. This may demonstrate that hospitals in these counties had already established a strong baseline in ensuring patient safety and successfully maintained this high performance.

It is necessary to discuss any significant results within the context of fluctuating surgical case volumes. In a 2021 national study, all major surgical procedure categories, except ear, nose, and throat (ENT) procedures, returned to pre-COVID-19 pandemic levels by late 2020 [4]. This rebound in surgical volumes was partly due to the scaling back of government mandates on surgical activity, which allowed hospitals to address the backlog of surgical procedures accordingly. Interestingly, this recovery of volumes occurred even in areas with high COVID-19 case counts and did not dip with subsequent COVID-19 surges, suggesting that individual hospitals developed their own frameworks for balancing public health risks with the growing demand for surgical care. By late 2020, many systems resumed outpatient procedures without compromising hospital bed availability for potential COVID-19 patients [4].

In Texas specifically, the COVID-19 surge in the summer of 2020 led to a series of executive orders issued by the governor, reinstating and repeatedly expanding the mandated postponing of elective surgeries by county [19]. By September 2020, the governor issued another executive order allowing for the elective surgery ban to be selectively lifted in areas with COVID-19 hospitalizations under 15% of hospital capacity [20]. The order also required that every hospital reserve at least 10% of its capacity for COVID-19 patients at various levels of severity to anticipate further surges [20]. While this move aimed to protect the availability of hospital beds, intensive care unit (ICU) beds, personal protective equipment (PPE) supplies, and staff, it had unintended consequences, particularly in rural regions, of cutting off a significant source of hospital revenue [21]. The subsequent financial strain, combined with rising labor and supply costs, placed additional pressure to address surgery backlogs [21]. One study performed predictive modeling to estimate how long it would take for backlogged surgeries to be addressed. Regarding recovery from a 12-week disruption in surgical services caused by COVID-19, projections suggest it would take a median of 45 weeks to eliminate the backlog if surgical capacity were raised by 20%. A smaller 10% increase would extend the timeline to around 90 weeks, while a 30% boost could shorten it to approximately 30 weeks [22].

When using the 2019 to 2022 raw data from the Texas Hospital Dataset and comparing the number of surgical procedures to hospital beds, Victoria County had several surgical procedure-to-hospital ratios of 14.7 in 2019, which steadily increased to 18.4 in 2022. Colorado County had a ratio of 50.5 in 2019 that peaked in 2020 at 52.8 before steadily falling to 51.2 [10]. Data from the Texas Department of State and Health Services on COVID-19 hospitalizations showed that between April 2020 and May 2023, the area containing Victoria County had 31 weeks of COVID-19 hospitalizations above 15% of capacity, while the area containing Colorado County had a little over 22 weeks [23]. In this context, it could be presumed that Victoria County hospitals had accumulated a higher burden of surgery backlogs to address and potentially a subsequent higher financial strain. Regarding the observed differences in Colorado and Victoria counties, it is hypothesized that Colorado County hospitals successfully adapted their post-pandemic policies. In contrast, Victoria County failed to adapt while simultaneously pushing to increase surgical capacity. The isolated significance observed in Colorado and Victoria counties further emphasizes the importance of local context, including variation in COVID-19 volume, procedural volume, hospital infrastructure, and financial strain. While Victoria’s increase in RSBs may reflect difficulties in managing post-pandemic surgical surges, Colorado’s decrease suggests successful long-term adjustments to surgical workflow. Overall, the minimal fluctuation in RSB rates across other Texas counties points to a broader capacity for health systems to maintain surgical safety even under crisis conditions.

A limitation of this study is that the dataset and subsequent analyses were dependent on the variables reported through the DRA-HAC measures, which are limited in scope and depth. These metrics only capture certain hospital-acquired conditions [10] and may not reflect the full clinical complexity of surgical environments or capture all instances of RSBs, particularly in facilities with inconsistent or incomplete reporting. Additionally, the data used were formatted in quarter-years rather than in exact annual increments, which limited the ability to identify trends aligned precisely with pandemic phases or policy changes. This format may also have obscured fluctuations in RSB rates that occurred within shorter time frames. Another important limitation is the potential for selection bias, as data with reported discrepancies was excluded from the dataset [10], and there is a chance that higher-functioning or more organized institutions had a higher capacity to report data and do so without error. This introduces a risk that the dataset may disproportionately reflect better-resourced hospitals, limiting the representativeness of the findings. Furthermore, generalizability is constrained by the study's focus on Texas hospitals and Medicare beneficiaries. The patterns observed may not apply to hospitals in other states with different healthcare systems, regulations, or resource availability, nor to younger or privately insured patient populations who may face different procedural volumes, follow-up patterns, or risk profiles. Future studies would benefit from more comprehensive data collection, analysis of hospital-specific resources (e.g., staffing, PPE, finances), and the integration of qualitative data about local policies (e.g., mask mandates, vaccine mandates), institutional policies, and surgical practices to provide a fuller understanding of what impacts surgical safety under crisis conditions.

## Conclusions

This study found no significant difference between the mean rates of RSB in pre-COVID versus post-COVID eras and by HHS region during this time. Moreover, all but two Texas counties (i.e., Victoria and Colorado) had no statistically significant difference between the mean rates of RSB. Given the importance of reducing and preventing RSBs, follow-up studies that review specific surgical protocols and policies before and after the COVID-19 pandemic in Victoria and Colorado counties should be conducted. By further investigating the factors that influenced these differences, there is hope to adjust healthcare facilities in other Texas counties to decrease the rate of or prevent a rise in RSBs and adverse patient safety outcomes during times of uncertainty.
